# How to Walk and Sit

**Published:** 1890-05

**Authors:** 


### HOW TO WALK AND SIT.

In the course of a lecture recently delivered in connection with the Jenness–Miller School for Physical Culture, in Hardman Hall, New York city, Miss Jenness showed her audience how to walk and how to sit. "In walking," she said, "the weight should be borne on the ball of the foot, both heel and ball coming down together. The chest should be ahead of the foot, if one stops suddenly. The toes should be turned out, and the heels should follow each other in a straight line."

Miss Jenness then gave several laughable illustrations of the way women walk. She followed this with similar descriptions of the way in which they sit. "A position which I constanly see," she said, "and it is taken by ladies who credit themselves with being well bred, is the one where one knee is crossed over the other. Such a position is indelicate; besides, I believe it produces paralysis. Never put one foot over the other, either; it is not graceful. It is not necessary to sit upright, but one should sit on the hips."

Miss Jenness next illustrated the incorrect positions women take in going up and down stairs. "In these motions," said she, "the weight must be placed on the ball of the foot and the chest should be raised. There is one strong point, however, to remember at all times, keep the chest up."

